# Challenges and prospects of *CYP2C19* genotype-guided clopidogrel therapy from the perspective of precision medicine

**DOI:** 10.3389/fphar.2026.1711350

**Published:** 2026-03-24

**Authors:** Zixiao Wang, Xiangkai Fu, Guoying Zhang

**Affiliations:** 1 The First Affiliated Hospital of Hebei North University, Zhangjiakou, China; 2 The First Hospital of Jilin University, Changchun, China; 3 Cangzhou Central Hospital, Cangzhou, China

**Keywords:** antiplatelet therapy, coronary arterydisease, CYP2C19 genotyping, pharmacogenomics, precision medicine

## Abstract

In the era of precision medicine, dual antiplatelet therapy with aspirin and a *P2Y12* receptor inhibitor is the standard strategy for patients after percutaneous coronary intervention (PCI). Clopidogrel, as a widely used representative drug, has its efficacy significantly influenced by *CYP2C19* genetic polymorphisms, leading to poor metabolism in some patients, insufficient exposure to active metabolites, and increased risk of ischemic events. In theory, *CYP2C19* genotyping to guide individualized antiplatelet therapy could help optimize the clinical selection of *P2Y12* inhibitors and improve prognosis. However, current evidence and guidelines do not support its use as a routine test for post-PCI patients, reflecting multiple challenges in clinical translation. From the perspective of precision medicine, this article systematically reviews the main problems and clinical application dilemmas of *CYP2C19* genotyping in guiding clopidogrel therapy, including controversies and uncertainties in randomized controlled trials (RCTs) evidence, neglect of rare alleles, complexity of metabolism regulated by polygenic and clinical factors, and limited predictive value for low platelet reactivity. Finally, this paper prospects future research directions and clinical translation prospects, aiming to provide a theoretical reference for promoting individualized antiplatelet therapy and balancing thrombosis and bleeding risks.

## 1 Background

In recent years, global changes in diet and lifestyle have led to an earlier onset of atherosclerotic cardiovascular disease (CVD), with its prevalence and mortality continuing to rise, making it a major public health burden (Le et al., 2024). Contemporary drug therapy is undergoing a profound transformation from traditional empirical mode to precision medical paradigm (Naithani et al., 2021). The core purpose of precision medicine is to integrate individual multi-dimensional biological information (especially genomic data), realize personalized optimization of disease prevention, diagnosis and treatment strategies, and ultimately improve curative effect and reduce treatment-related risks (Sisodiya, 2021). In this transformation, pharmacogenomics, as its key pillar discipline, systematically studies the influence mechanism of genetic variation on individual differences in drug responses. It explains how the sequence polymorphism of drug metabolizing enzymes, transporters, action targets and related pathway genes regulate the pharmacokinetics and pharmacodynamics of drugs, and provides genetic basis for making individualized drug administration plans in clinic (Caudle et al., 2025). Therefore, pharmacogenomics is not only a bridge between gene information and clinical decision-making, but also a core scientific tool to realize the vision of precision medicine.

**FIGURE 1:**
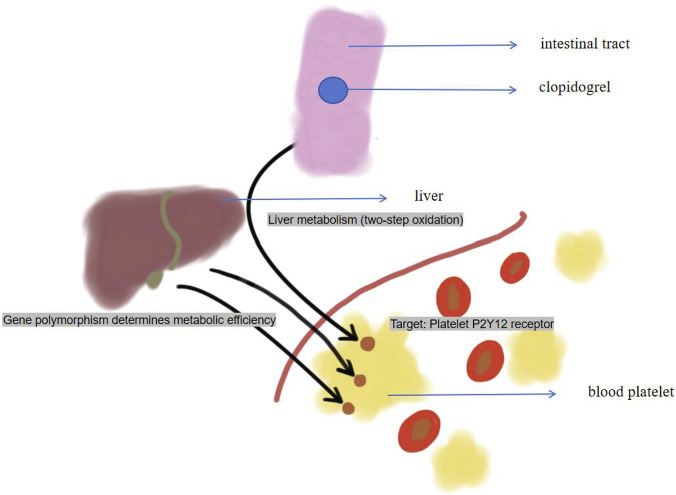
clopidogrel metabolism and *CYP2C19*.

The clinical application of clopidogrel and its remarkable individual reaction difference provide a classic example for the principle of pharmacogenomics. Clopidogrel, a *P2Y12* receptor antagonist, is widely used in antiplatelet therapy for acute coronary syndrome (ACS) and after PCI, significantly reducing the risk of thrombotic events. However, despite its broad application, some patients still experience ischemic events such as stent thrombosis and recurrent myocardial infarction during clopidogrel treatment, raising widespread concern. In 2010, the U.S. Food and Drug Administration (FDA) issued a black box warning for clopidogrel, highlighting significant variability in its antiplatelet effects—particularly associated with loss-of-function (*CYP2C19*) alleles of *CYP2C19* that lead to poor metabolism (Figure 1). To address the clinical challenges posed by variable responses to clopidogrel, more potent third-generation *P2Y12* inhibitors, such as prasugrel and ticagrelor, have been introduced. Although these agents demonstrated superior efficacy in preventing ischemic events in randomized controlled trials, their broader application is limited by higher bleeding risk, lower cost-effectiveness, and non-hemorrhagic side effects (e.g., ticagrelor-associated dyspnea) (Pereira et al., 2024). According to European multicenter registry data, clopidogrel remains the maintenance antiplatelet therapy in 35%–55% of patients at discharge (Tscharre et al., 2017).

In this context, *CYP2C19* gene polymorphism is a key pharmacogenomic marker for advancing personalized antithrombotic therapy. Detecting poor metabolizers through genotyping may facilitate treatment strategy switching, thereby potentially improving clinical outcomes. However, the application of this approach in current clinical practice still faces multiple challenges, including controversies and uncertainties in RCTs evidence, neglect of rare alleles, complexity arising from the interplay of polygenic and clinical factors regulating metabolism, and limited predictive value for low on-treatment platelet reactivity. From a precision medicine perspective, this review systematically examines the key issues and implementation barriers associated with the use of *CYP2C19* genotyping to guide clopidogrel therapy, and explores future directions, aiming to provide an evidence-based foundation and theoretical framework for optimizing antiplatelet strategies and balancing ischemic and bleeding risks.

## 2 Precision medicine for *CYP2C19* genetic polymorphisms and randomized controlled trials (RCTs)

Multiple RCTs investigating the clinical benefits of *CYP2C19* genotype-guided *P2Y12* inhibitor therapy have yielded markedly controversial and inconsistent results, underscoring the persistent uncertainty surrounding its applicability in clinical practice (Table 1).

**TABLE 1:** Main RCTs to evaluate the selection of *P2Y12* inhibitors guided by *CYP2C19* genotype.

Randomized trial	Sample size	Region	Population	Endpoint events	Follow-up duration	Results	Conclusion	Limitations
Single-center randomized trial	600	China	CAD patients	Death, myocardial infarction (MI), stroke or ischemia-driven target-vessel revascularization	180 days	At 180 days, the genotype-guided group had 3 cases of death, MI, or stroke compared to 18 cases in the control group (1.0% vs. 6.2%, P < 0.01)	Individualized antiplatelet therapy based on CYP2C19 genotyping significantly reduced major adverse cardiovascular events without increasing bleeding	In a single center study in Chinese, the sample size was too small, and CYP2C19*17, ABCB1 and ITGB3 genes were not detected
Single-center randomized trial	628	China	CAD patients	All-cause death, MI or target vessel revascularization	12 months	At 1, 6, and 12 months, the MACE incidence was significantly lower in the genotype-guided group than in the control group (1.3% vs. 5.6%, P = 0.003; 3.2% vs. 7.8%, P = 0.012; 4.2% vs. 9.4%, P = 0.010). Bleeding rates were not significantly different (P > 0.05)	Individual antiplatelet therapy guided by CYP2C19 genetic testing significantly reduced the rate of MACE without an increase in the rate of bleeding	The risk of selection bias in single-center research; The confounding factors of platelet reactivity are not completely controlled (such as diabetes, age, BMI and other known confounding variables); key clinical endpoints (such as in-stent restenosis and stent thrombosis) were not covered
ONSIDE TEST study	94	Poland	Stable CAD patients	Incidence of periprocedural myocardial injury	12 months	The incidence of periprocedural myocardial injury was similar in the genotype-guided and control groups after 1 year (76.5% vs. 73.1%)	Early prasugrel therapy guided by genotyping may reduce the extent of myocardial injury during PCl in stable CAD patients	Small sample size, Given the specific inclusion and exclusion criteria, selection bias cannot be ruled out
POPular Genetics study	2488	Europe (10 sites)	STEMI patients	All-cause death, MI, definite in-stent thrombosis, stroke, major bleeding	12 months	The main comprehensive results occurred in 63 genotype-guided patients and 73 control patients (5.1% vs. 5.9%, P < 0.001).Compared with the control group,the bleeding events in genotype guidance group were significantly reduced (9.8% vs. 12.5%, P = 0.04)	Genotype-guided therapy was non-inferior to prasugrel/ticagrelor for thrombotic events and had lower bleeding risk	The confounding factors affecting the endpoint events are not completely controlled; the CYP2C19*17, ABCB1 and ITGB3 genes were not analyzed
PHARMCLO trial	888	Europe	ACS patients	Composite of cardiovascular death, non-fatal MI, non-fatal stroke, and major bleeding	12 months	The endpoint events occurred in 71 patients in genotype guidance group (15.9%)and 114 patients in standard treatment group (25.9%, P < 0.001)	CYP2C19 gene detection individualized antiplatelet therapy can reduce ischemic and bleeding events	Early termination of the experiment will ffect the validity and generalization of the results
TAILOR-PCI randomized trial	5302	USA, Canada, korea, Mexico	ACS and stable CAD patients	Composite endpoint of cardiovascular death, MI, stroke, stent thrombosis and severe recurrent ischemia	12 months	The primary endpoint occurred in 113 genotype-guidedCompared with the standard clopidogrel treatment, severe recurrent ischemia.patients (4.4%) vs. 135 conventional patients (5.3%, P = 0.16)	Compared with the standard clopidogrel treatment,genotype-guided treatment did not observe a significant reduction in ischemic events	Insufficient statistical efficiency caused by sample size design; actual treatment compliance may affect the outcome

Several single-center studies have suggested potential clinical benefits of a genotype-guided strategy. One RCT involving 600 Chinese PCI patients demonstrated that antiplatelet therapy adjustment based on *CYP2C19* genotype—including standard or doubled clopidogrel doses, or cilostazol combination—significantly reduced ischemic events within 180 days without increasing bleeding risk (Xie et al., 2013). Similarly, another single-center study of 628 Chinese patients found that the genotype-guided group (receiving clopidogrel or ticagrelor based on metabolic phenotype) had significantly lower composite endpoint events within 12 months compared to the conventional clopidogrel group, with no difference in bleeding risk (Shen et al., 2016). However, these studies have notable limitations: small sample sizes, restriction to specific ethnic populations (e.g., Chinese), and failure to include other genetic variants such as CYP2C19*17, *ABCB1*, or *ITGB3* that may influence clopidogrel response, thereby limiting the generalizability of their conclusions. Furthermore, the ONSIDE TEST study, which enrolled 94 patients with stable coronary artery disease, found no significant difference in perioperative myocardial injury between the genotyping group and the control group, further underscoring the uncertainty regarding the benefits of genotype guidance in small-scale studies (Tomaniak et al., 2017).

The results of several large multicenter RCTs have intensified the controversy. The POPular Genetics trial, which included 2,488 ST-segment elevation myocardial infarction (STEMI) patients, demonstrated that a genotype-guided selection of *P2Y12* inhibitors—where *CYP2C19* allele carriers received ticagrelor or prasugrel and non-carriers received clopidogrel—was non-inferior to standard potent *P2Y12* inhibitor therapy regarding thrombotic risk at 12 months, while significantly reducing bleeding events (Claassens et al., 2019). In contrast, the PHARMCLO trial utilized a strategy combining multi-gene profiling of *ABCB1* and CYP2C19*2/*17 with clinical factors to guide treatment, and reported significantly fewer composite endpoint events in the genotype-guided group compared to conventional treatment. However, this trial was terminated prematurely and enrolled only approximately 25% of the planned sample size, resulting in a substantial risk of bias (Notarangelo et al., 2018). Conversely, the TAILOR-PCI trial, a large RCT involving 5,302 patients, found no significant difference in ischemic endpoints at 12 months between the genotype-guided strategy, in which loss-of-function carriers received ticagrelor and non-carriers received clopidogrel, and the conventional clopidogrel group, thus failing to establish the superiority of genotype-guided therapy in preventing ischemic events (Pereira et al., 2020).

These studies share several methodological limitations that warrant cautious interpretation. First, the enrolled populations were highly heterogeneous, encompassing different clinical presentations (e.g., ACS, stable coronary artery disease, STEMI) and multi-national cohorts. Second, high-quality comparative data on alternative antiplatelet regimens for *CYP2C19* allele carriers—particularly in Asian populations—remain scarce. Finally, most studies had relatively short follow-up periods, and large randomized trials such as TAILOR-PCI reported neutral outcomes, highlighting the current limitations of genotyping strategies. Future research should incorporate more comprehensive genetic testing (e.g., including CYP2C19*17, *ABCB1*, and other relevant polymorphisms) and extended follow-up to better evaluate the potential benefits of genotype-guided therapy.

## 3 Distribution and pharmacogenomics detection of *CYP2C19* gene polymorphism in different populations

Currently, *CYP2C19* genotyping assays used in clinical practice are primarily designed to identify known common alleles (e.g., *2 and *3), rather than to detect novel or rare variants. Consequently, individuals carrying rare loss-of-function variants (e.g., CYP2C19*4 and *8) may be misclassified as normal metabolizers (*1/*1) due to lack of coverage in standard testing panels (Lee et al., 2022). Such misclassification can lead to deviations in genotype-guided antiplatelet therapy, for example, when intermediate or poor metabolizers who should avoid clopidogrel are incorrectly recommended to receive standard-dose treatment.

The risk of such misjudgment varies according to the distribution of *CYP2C19* genotype and phenotype in different populations (Table 2): the frequency of *CYP2C19*2 and three alleles is higher in the indigenous population of East Asia and Oceania, and the slow metabolism (PM) phenotype is higher; The CYP2C19*17 allele is more common in European, Middle Eastern and American populations, and the ultra-fast metabolism (UM) phenotype is more common. The distribution of alleles and phenotypes in African population is highly heterogeneous, while the mixed-race population in America presents mixed characteristics between Europe, Africa and indigenous people. The allele *4 is not uncommon in Latin America (0.8%–1.2%) and some South Asian/African populations (1%–2%), and the frequency of *8 in African populations can reach 0.5%–1%, which highlights the deficiency of the detection strategy in population applicability.

**TABLE 2:** Comparison table of *CYP2C19* genotype and phenotype.

Population	CYP2C19*1	CYP2C19*2	CYP2C19*3	CYP2C19*17	CYP2C19*1/*1	CYP2C19*1/*2
Caucasian	69.60%	13.20%	0.40%	15.80%	48.00%	23.00%
African	51.00%	17.00%	N	22.00%	N	N
African-American	59.40%	19.40%	0.40%	18.20%	35.00%	29.00%
Hispanic	70.40%	12.80%	0.00%	15.20%	50.00%	22.00%
Indian	42.00%	40.20%	0.00%	17.80%	16.10%	31.00%
Han Chinese	56.20%	38.6%	5.20%	N	35.00%	38.00%
Japanese	57.90%	27.90%	12.80%	1.30%	35.50%	N
Malaysian	80.70%	5.70%	6.50%	4.80%	66.10%	9.70%
Thais	70.14%	25.36%	4.50%	N	48.82%	36.02%
Iran	86.00%	13.00%	1.00%	N	74.00%	24.00%

More importantly, rare and low-frequency variations are probably the key components of the “lack of heritability” of clopidogrel reaction. Common single nucleotide polymorphisms (SNP) on which genome-wide association studies (GWAS) depends can only explain some genetic variations. More and more evidences show that rare variations, especially those functional variations with moderate to large effect scale, significantly affect *CYP2C19* enzyme activity, clopidogrel metabolism and platelet inhibition effect through accumulation or synergy. Therefore, it is difficult for the prediction model based on common variation to fully capture the reaction differences among individuals.

Functional interpretation of rare gene variants is the premise of understanding the clinical significance of rare variants, and sequencing technology is the basis of discovering these variants. Whole Exon Sequencing (WES) has become the main tool to screen the rare variation of coding region because of its cost-effectiveness and target area coverage efficiency. Whole genome sequencing (WGS) can further reveal the non-coding regulatory regions and structural variations. A study combining *in vitro* experiments and computational simulations shows that rare mutations such as p. Ala297Val and p. His251Gln can make *CYP2C19* enzyme activity lose more than 97%, and if its function is ignored in clinical tests, it will directly lead to serious treatment decision-making mistakes (Johansson et al., 2025). In summary, current genotyping strategies centered on common variants exhibit clear limitations in both coverage and genetic explanatory power.

Integrating rare variants into routine genotyping panels remains challenging, especially in populations where allele frequencies are extremely low, and the balance between detection costs and clinical benefit has yet to be established. Existing gene testing approaches require further optimization to improve the comprehensiveness and accuracy of phenotype prediction while maintaining clinical utility.

## 4 Polygenic and clinical factors jointly determine clopidogrel metabolism

While *CYP2C19* genetic polymorphism is a key factor influencing the metabolic activation and antiplatelet effects of clopidogrel, it represents only one component within a complex metabolic pathway. The variability in clopidogrel response arises from the combined effects of polygenic background and clinical environmental factors. In precision medicine practice, reliance solely on *CYP2C19* genotype is often insufficient to comprehensively predict individual treatment outcomes.

Beyond *CYP2C19*, multiple genes involved in the pharmacokinetic and pharmacodynamic processes of clopidogrel can be categorized into three groups (Jiang et al., 2015): those related to intestinal absorption (e.g., *ABCB1*), hepatic metabolic activation (e.g., *CYP2C19*, *CES1, PON1*, and *B4GALT2*), and interaction with platelet surface receptors (e.g., *P2Y12, ITGB3*, and *GPIIb/IIIa*). Several studies suggest that polymorphisms in genes such as *ABCB1, CES1, ITGB3*, and *PON1* may also be associated with clopidogrel’s antiplatelet effects and clinical endpoints (Castrichini et al., 2023). For instance, a study involving approximately 18,500 PCI patients demonstrated that genetic variants in *CYP2C19*, *ABCB1*, and *ITGB3* were significantly correlated with early stent thrombosis (Díaz-Villamarín et al., 2016). *CES1,* responsible for hydrolyzing clopidogrel into inactive metabolites, may─through functional variants─alter the generation of active metabolites and thereby interfere with antiplatelet efficacy (Duarte and Cavallari, 2021).

It is noteworthy that pleiotropy significantly increases the complexity of predicting clopidogrel response. Accumulating evidence indicates that variants at a single genetic locus can influence the pharmacokinetics and pharmacodynamics of clopidogrel through distinct yet synergistic biological pathways. For instance, in addition to its primary role in hepatic bioactivation, certain *CYP2C19* variants have been associated with pathophysiological processes such as platelet activation and endothelial function. These parallel pathways may collectively modulate the final antiplatelet effect. In response to this intricate network, several studies have validated the clinical relevance of pleiotropy using polygenic models. For example, research conducted in a Puerto Rican population demonstrated that variants in genes other than *CYP2C19*, such as *CES1* and *B4GALT2*, independently and synergistically affect levels of clopidogrel’s active metabolite and platelet reactivity (Claudio-Campos et al., 2015). This study further developed a polygenic risk score model integrating multi-locus genetic information from *CYP2C19*, *CES1*, and *ABCB1*, which showed significantly superior predictive performance compared to single-genotype models. These findings underscore the necessity of comprehensively evaluating polygenic interactions within specific populations.

In addition to genetic background, various clinical and demographic factors significantly affect clopidogrel treatment efficacy. The ABCD-GENE score integrates nongenetic variables─such as age, body mass index, diabetes, and chronic kidney disease─with *CYP2C19* genotype to identify patients at high risk for high on-treatment platelet reactivity (Angiolillo et al., 2020). Furthermore, ethnicity, sex, obesity, comorbidities, and drug-drug interactions (e.g., concomitant use of proton pump inhibitors or *CYP2C19* inhibitors) may modulate clopidogrel metabolism and response pathways, further contributing to the complexity of interindividual variability (Lee et al., 2020). When considering the difference of drug reaction, ethnic background must be taken as the key covariate in the analysis. There are systematic differences in genetic characteristics, allele frequency and environmental exposure among different populations, which may lead to the limited applicability of gene-phenotype association based on single population data in Latin America, Africa or Asia. In addition, in clinical practice in specific regions (such as Latin America), non-biological factors such as drug accessibility, treatment cost, medical insurance policy and local prescription habits often become major practical constraints affecting the selection of new *P2Y12* inhibitors. Therefore, when formulating regional treatment strategies, it is necessary to integrate population-specific pharmacogenomic evidence with local health system and economic reality to avoid the disconnection between guidelines and clinical practice (Yang et al., 2025).

## 5 Limited predictive value of *CYP2C19* genotyping for low on-treatment platelet reactivity

Within the framework of precision medicine, *CYP2C19* genotyping holds significant value for identifying individuals with abnormal responses to clopidogrel therapy. However, its clinical application faces a dual challenge: effectively recognizing high ischemic risk while also carefully assessing elevated bleeding risk.

Extensive studies have confirmed a strong association between *CYP2C19* alleles and high on-treatment platelet reactivity (HTPR) following clopidogrel administration (Seip et al., 2010). For instance, the GEPRESS study—a prospective, multicenter observational investigation involving 1,053 patients with non-ST-elevation myocardial infarction—identified CYP2C19*2 as the single nucleotide polymorphism most significantly correlated with HTPR (Palmerini et al., 2014). A meta-analysis of 4,341 patients receiving a 600 mg clopidogrel loading dose further demonstrated that carriers of *CYP2C19* alleles exhibited significantly higher residual platelet reactivity compared to non-carriers. Given the established link between HTPR and increased risk of ischemic events after PCI, HTPR is widely regarded as a surrogate endpoint for adverse cardiovascular outcomes, and *CYP2C19* genotyping offers certain predictive value in identifying individuals at high ischemic risk (Galli et al., 2021).

However, variability in clopidogrel response encompasses not only HTPR but also low on-treatment platelet reactivity (LTPR). A meta-analysis of 20 studies involving 19,064 patients indicated that LTPR is significantly associated with an increased risk of bleeding following coronary stent implantation (Bálint et al., 2023). It is noteworthy that *CYP2C19* genotype has limited predictive power for LTPR, particularly in populations at low ischemic risk. Currently, there is insufficient evidence to support that routine genetic testing provides net clinical benefit in this subgroup.

## 6 Clinical implementation suggestions and future directions for *CYP2C19* genotyping

The selective use of *CYP2C19* genotyping by clinicians in high-risk subgroups requires comprehensive consideration of multiple factors. Current evidence indicates that genotype-guided therapy reduces the risk of major adverse cardiovascular events by 31% in ACS patients undergoing PCI, particularly in East Asian populations (where the carrier rate of *2/*3 alleles is approximately 30%) (Huang et al., 2023; Angiolillo et al., 2024). In clinical practice, testing is recommended preferentially for patients with high-risk features, including a history of stent thrombosis, diabetes mellitus, chronic kidney disease, or STEMI treated with PCI (Kheiri et al., 2019; Hulot et al., 2020). Rapid point-of-care testing is the preferred strategy, capable of returning results within 2.1 h (Bergmeijer et al., 2018).

Integrating *CYP2C19* genotyping into a broader disease risk management framework—rather than considering it in isolation for cardiovascular disease—may better reflect its clinical value. For example, a single genetic test result can inform not only antiplatelet therapy but also the use of other medications such as proton pump inhibitors and antidepressants. This cross-indication synergy should be considered in clinical decision-making (Chetty et al., 2021).

Numerous studies have confirmed that a combined strategy integrating both genetic testing and platelet function assays is significantly superior to either method alone in reducing the incidence of major adverse cardiovascular events (MACE), without increasing the risk of bleeding. This benefit is particularly pronounced in patients undergoing complex percutaneous coronary intervention (PCI) and those at high thrombotic risk (Kim et al., 2023; Galli et al., 2023). Furthermore, the dual testing approach demonstrates considerable potential for guiding short-term (1-year) individualized antiplatelet therapy following PCI for coronary artery disease, effectively reducing ischemic events within the first year without elevating the risk of major bleeding (Yu et al., 2022). Furthermore, such a multidimensional strategy not only identifies high-risk patients who might be missed by genetic testing alone, such as *CYP2C19* normal metabolizers with clopidogrel hyporesponsiveness, but also supports precision treatment across different metabolic phenotypes.

The development of integrated polygenic risk assessment models represents a major future direction for advancing clopidogrel personalization. Beyond *CYP2C19*, polymorphisms in genes such as *ABCB1, PON1, CES1,* and *B4GALT2* collectively influence clopidogrel pharmacokinetics and pharmacodynamics. Future research should focus on incorporating these genetic signals into unified algorithms to establish more comprehensive predictive models. For example, patients with both *CYP2C19* intermediate metabolism and *ABCB1 TT* genotype may face higher thrombotic risk than those with either variant alone. Although such polygenic risk scores require validation through large-scale randomized trials, they hold promise for significantly improving risk stratification accuracy and reducing false-positive or false-negative results associated with single-gene testing.

Furthermore, the clinical application of ticagrelor and prasugrel is significantly constrained in many low- and middle-income regions, including Latin America. Key limitations encompass: barriers to accessibility (limited inclusion in public health system formularies and unstable supply chains), high costs and restricted insurance coverage (leading to substantial out-of-pocket expenses and reduced treatment adherence), and clinical practice inertia (persistent physician reliance on conventional clopidogrel). Consequently, translating evidence-based optimal therapy into practice in these settings requires not only individualized risk stratification for high-risk patients but also systemic improvements in health policy. Such measures may include price negotiations and insurance reforms to enhance drug availability, alongside the generation of local real-world evidence to inform treatment strategies aligned with regional realities.

## 7 Conclusion

Within the framework of precision medicine, guiding clopidogrel therapy through *CYP2C19* genotyping faces multiple challenges, including controversies and inconsistencies in RCT evidence, underdetection of rare alleles, complexity arising from the interplay of polygenic and clinical factors, and limited predictive value for low on-treatment platelet reactivity. Nevertheless, it remains a promising direction for advancing individualized antiplatelet treatment. Through multidisciplinary collaboration, technological innovation, and the accumulation of real-world evidence, it is anticipated that current limitations can be progressively overcome, ultimately leading to safer and more effective therapeutic strategies for patients. This endeavor requires concerted efforts from researchers, clinicians, patients, policymakers, and industry stakeholders to jointly promote the development of precision medicine in clinical practice.
